# Living with a large predator: Assessing the root causes of Human–brown bear conflict and their spatial patterns in Lahaul valley, Himachal Pradesh

**DOI:** 10.1002/ece3.9120

**Published:** 2022-07-17

**Authors:** Vineet Kumar, Amira Sharief, Ritam Dutta, Tanoy Mukherjee, Bheem Dutt Joshi, Mukesh Thakur, Kailash Chandra, Bhupendra Singh Adhikari, Lalit Kumar Sharma

**Affiliations:** ^1^ Zoological Survey of India Kolkata India; ^2^ Wildlife Institute of India Dehradun India

**Keywords:** conflict mitigation, human‐brown bear conflict, India, SaTScan, trans–Himalaya

## Abstract

Brown bear‐mediated conflicts have caused immense economic loss to the local people living across the distribution range. In India, limited knowledge is available on the Himalayan brown bear (HBB), making human–brown bear conflict (HBC) mitigation more challenging. In this study, we studied HBC in the Lahaul valley using a semi‐structured questionnaire survey by interviewing 398 respondents from 37 villages. About 64.8% of respondents reported conflict in two major groups—crop damage (30.6%) and livestock depredations (6.2%), while 28% reported both. Conflict incidences were relatively high in summer and frequently occurred in areas closer to the forest (<500 m) and between the elevations range of 2700 m to 3000 m above sea level (asl). The dependency of locals on forest resources (70%) for their livelihood makes them vulnerable to HBC. The “upper lower” class respondents were most impacted among the various socioeconomic classes. Two of the four clusters were identified as HBC hot spots in Lahaul valley using SaTscan analysis. We also obtained high HBC in cluster II with a 14.35 km radius. We found that anthropogenic food provisioning for HBB, livestock grazing in bear habitats, and poor knowledge of animal behavior among the communities were the major causes of HBC. We suggest horticulture crop waste management, controlled and supervised grazing, ecotourism, the constitution of community watch groups, and others to mitigate HBC. We also recommend notifying a few HBB abundant sites in the valley as protected areas for the long‐term viability of the HBB in the landscape.

## Graphical abstract

1. About 64.8% of respondents reported conflict into two major groups ‐ crop damage (31.4%) and livestock depredations (6.2%) and were relatively high in summer and frequently occurred in areas closer forest between the 2700m to 3000m asl. We identified four clusters of HBC, and the hotspots lies in clusters 2 and 4. #10; #10;2. We found that anthropogenic food provisioning of bears, livestock grazing in bear habitats and poor knowledge of animal behaviour among the communities are the major causes of HBC. #10;3. We suggest proper disposal of horticulture crop waste, mass awareness among communities, controlled and supervised grazing as an immediate management strategy to reduce the HBC.

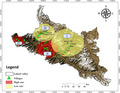

## 1 INTRODUCTION

There is a general agreement that biodiversity is under threat globally due to human population growth and the exponential use of natural resources (Blount‐Hill, 2021; Whittaker et al., 2005; Zarzo‐Arias et al., 2021). The human population expansion into the wildlife habitat, agricultural intensification, change in the cropping pattern, and dependence on natural resources are the main causes behind the widespread human–wildlife conflicts (Berihun et al., 2016; Dickman, 2010; Lozano et al., 2019; Miller, 2015; Sharma et al., 2021; Treves & Karanth, 2003). The brown bear (*Ursus arctos* Linnaeus, 1758) is distributed in most of Europe, Asia, North America, the Middle East, and some parts of North Africa (Servheen et al., 1999; Swenson et al., 2000). According to the global assessment, the brown bear is categorized as “Least Concern” by International Union for Conservation of Nature Red List (IUCN). However, the Himalayan brown bear (*Ursus arctos isabellinus* Horsfield, 1826) population is considered endangered as per the Red List criterion D. It occupies the Northern and Southern flanks of the Himalayas in Pakistan, Afghanistan, and India (McLellan et al., 2017; Nawaz, 2007; Sathyakumar, 2001). In the Indian Himalayan Region (IHR), HBB has a limited and narrow distribution range in the high elevation zones of Jammu and Kashmir Union territory (UT), Ladakh UT, Himachal Pradesh, and Uttarakhand (Sathyakumar, 2006). The existence of human–wildlife conflict is an undeniable challenge to wildlife conservationists. In recent years, HBC has increased globally and has become a major challenge for long‐term conservation and management (Dai et al., 2020; Xu et al., 2019). The movement and landscape utilization patterns of the bears are governed by food availability (Sharma, 2012; Soofi et al., 2018). The anthropogenic food near bear habitats leads to bear–human interactions throughout their distribution ranges. Such movements from natural habitats to croplands result in direct attacks on humans and crop damage (Bombieri et al., 2019; Hipolito et al., 2020; Krofel et al., 2020; Rathore, 2008). Moreover, several studies have suggested that livestock depredation by the bears is one of the major issues leading to aggravated human–bear conflicts (Goldstein, 2002; Goldstein et al., 2006; Kharel, 1997; Sekhar, 1998; Stein, 2000).

However, in India, studies on ecological aspects are limited, and most of them are of short terms conducted in selected landscapes (Nawaz, 2007; Rathore, 2008; Sharief et al., 2020). Furthermore, human–HBB interactions are widespread across the distribution range, but the negative interactions are putting the safety of humans and HBB at risk and leading to economic losses to the communities. The increasing economic loss and life threats to humans result in antagonistic behavior among the local communities toward the HBB. The retaliatory killing of the HBB by the migratory shepherd and local communities to reduce livestock depredation is a serious conservation and management challenge (Beckmann & Lackey, 2018; Rathore & Chauhan, 2014a; Sathyakumar, 2001). Previous studies from the other part of Himachal Pradesh brought out that crop damage by HBB is leading to economic losses to local communities (Rathore, 2008). The crop depredation by the black and HBB is more prevalent in the forest–village interface throughout the Western Himalayan region of India (Charoo et al., 2011; Chauhan, 2003). The HBB populations are mostly disjunct and are mostly restricted to protected areas (PA) distributed in the high elevation zones. Whereas, the HBB population in Lahaul valley of Himachal Pradesh occupies habitats outside the PA and in close proximity to the human settlement (Sharief et al., 2020). The increasing human interactions with the HBB lead to HBC, which is a serious threat to HBB's long‐term viability. Therefore, it is imperative to understand HBC in the Lahaul valley to develop data‐driven effective conflict mitigation strategy. Hence, we designed this study with an aim to document and analyze the nature, extent, and cause of human–HBB conflict. Furthermore, we also modeled the HBC hot spots in the region for better management of the HBC.

## 2 MATERIALS AND METHODS

### 2.1 Study area

**FIGURE 1:**
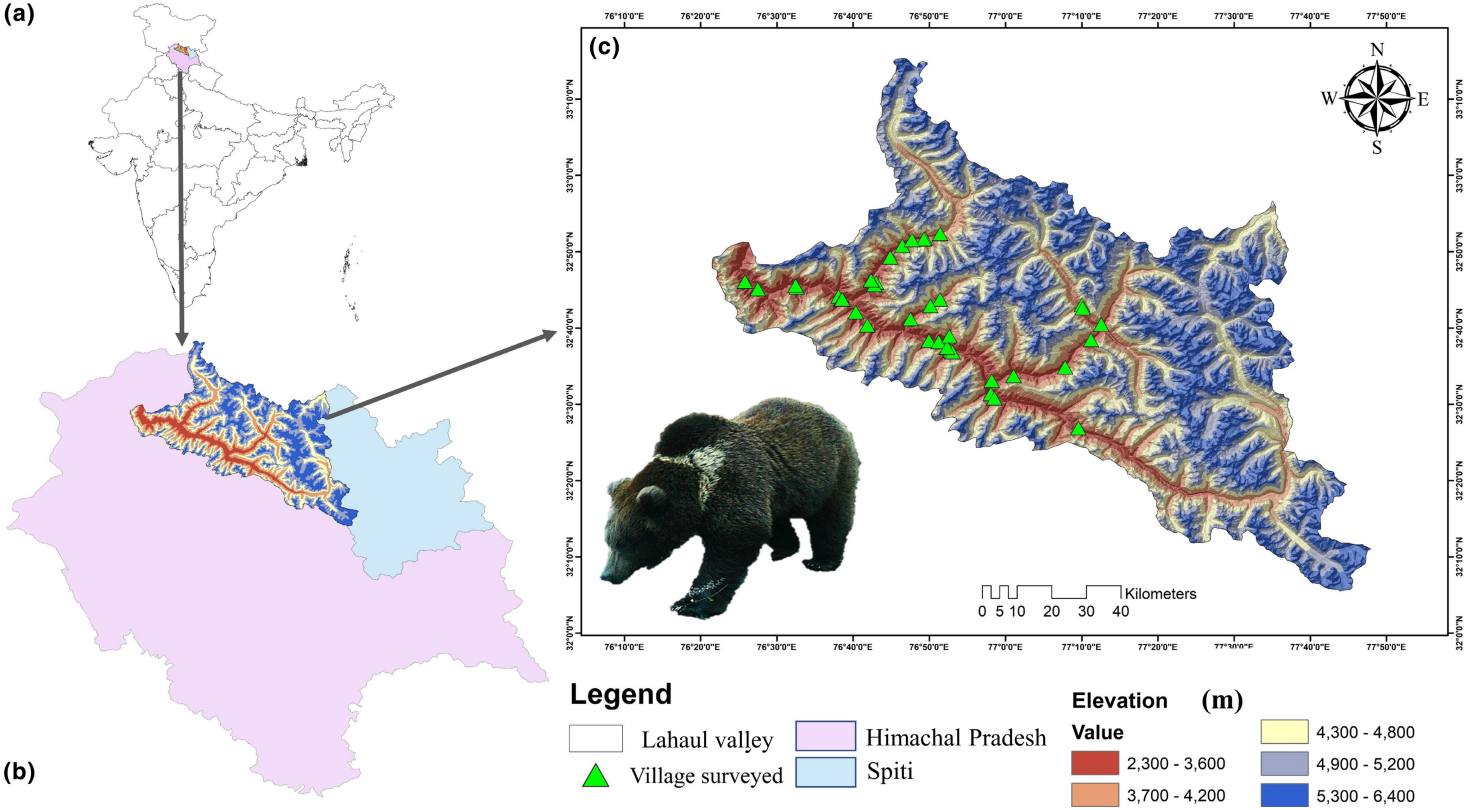
Map of study area. (a) showing the country boundary of India. (b) showing the position of Lahaul and Spiti district within state boundary of Himachal pradesh. (c) showing the study area (Lahaul Valley) where the elevation ranges depicted by color ramp

The study was carried out in Lahaul valley of district Lahaul & Spiti (L&S) of Himachal Pradesh, located from 31°44′57″ to 32°59′57″ N Lat and 76°46′29″ to 78°41′34″E long (Figure 1; Aswal & Mehrotra, 1994). The research permission was obtained from Principal Chief Conservator of Forest (Wildlife) ‐cum Chief Wildlife Warden, Himachal Pradesh Shimla‐700,053 (letter no. W.L./Research Study/W.L.M./2291) to conduct the survey. The topography of the study area is mountainous, comprising rugged deep gorges and steep slopes with an altitude ranging from 2400 to 6400 m. The valley has a temperate and alpine climate (Joshi et al., 2006), dominated by dry temperate to dry alpine type vegetation, one of the main habitats for the HBB in the valley (Joshi et al., 2020; Sharief et al., 2020). The livelihood for much of the local communities is agrarian, growing horticulture, and high market value vegetable crops. As the cropland of the valley is highly productive, both the local and the exotic vegetables are cultivated in the valley, which fetches high income for the local communities. In Lahaul valley, local/traditionally grown crops are maize (*Zea mays*), pea (*Pisum sativium*), potato *(Solanum tuberosum*), wheat (*Triticum aestivum*), barley (*Hordeum vulgare*), and buckwheat (*Fagopyrum esculentum*). Whereas, exotic/economic crops such as beans (*Phaseolus vulgaris*), iceberg lettuce (*Lactuca sativa*), cabbage (*Brassica oleracea var. capitata*), red leaf lettuce (*Lactuca Sativa Var. Crispa*), and broccoli (*Brassica oleracea var. italica*) are under cultivation since last one decade and used for both locals subsistence and market. However, among the horticultural crops, apple (*Malus domestica*) and apricot (*Prunus armeniaca*) are the most common orchards in the study area. Furthermore, animal husbandry is also followed by remote communities and also nomadic grazers visiting the highland grazing grounds from other regions of Himachal Pradesh. In addition, locals rear different livestock such as cattle, sheep, goat, mule, donkey, and domestic yak. Other than HBB, other large carnivores such as snow leopard (*Panthera uncia*), Himalayan wolf (*Canis lupus chanco*), and Himalayan red fox (*Vulpes vulpes*) are also distributed in the landscape. The dominant herbivores such as Himalayan ibex (*Capra sibrica*), Himalayan musk deer (*Moschus leucogaster*), and Himalayan thar (*Hemitragus jamlahicus*) also have viable populations in the landscape.

### 2.2 Methods

**FIGURE 2:**
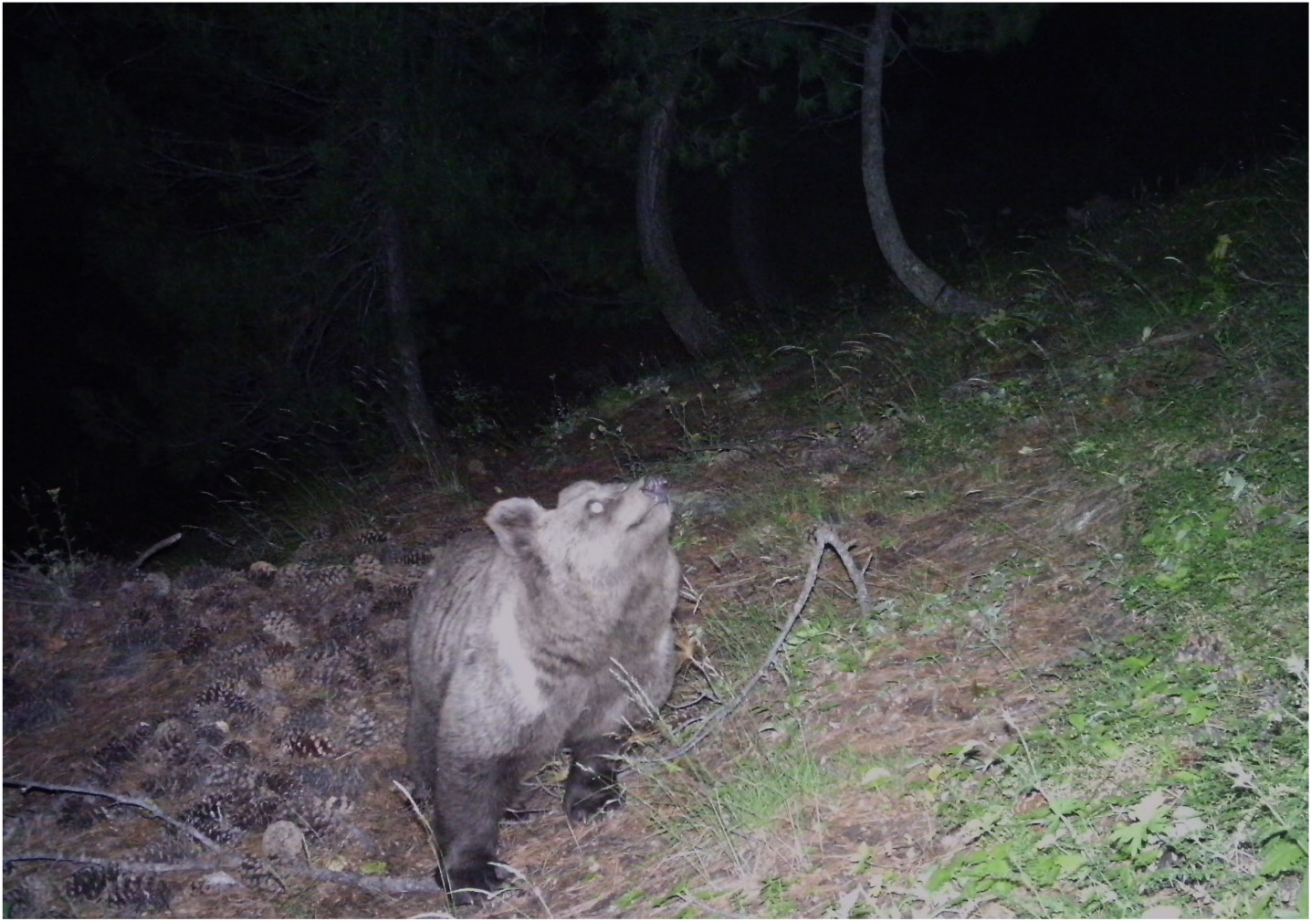
Showing the camera trap capture of Himalayan brown bear (*Ursus arctos isabellinus*) in Lahaul Valley landscape

The assessment of the human–HBB conflict was carried out in Lahaul valley from July 2018 to December 2019. We interviewed local communities using a semi‐structured “close and open‐ended” questionnaire to collect information on various aspects of human–HBB conflict in the study landscape (Temesgen et al., 2022). We also placed a total of twelve camera traps in the agriculture field and recorded the presence of HBB in the seven camera traps (Figure 2). We used a stratified random sampling approach (Taherdoost, 2016) to record the responses of locals from 37 villages distributed throughout the valley. About 30% of total households were interviewed from each village representing affluent and nonaffluent socioeconomic status following the National Sample Survey Organization guidelines, Government of India (Cochran, 1977; Joshi et al., 2020; Jyrwa et al., 2020), and NSSO, 2011). A total of 398 respondents (male = 294 and female = 104) were interviewed, and only one representative individual from each household, mainly the head of the family, was considered. We asked both open‐ and close‐ended questions to gather information on types and causes of conflicts, location, timing, seasonality, and place of conflict incidence. We have considered only one record of conflict presence irrespective of the number of conflict cases to maintain consistency and mitigate correlation. We further gathered information on the attitude of locals toward wildlife. The human–HBB conflict was categorized into three categories (a) crop damage (agricultural/horticultural), (b) livestock depredation, and (c) human attacks and casualties. The data were also gathered on mitigation measures used by the local communities to minimize conflicts with HBB. The GPS coordinates along with other covariates such as types and causes of conflict (crop damage or livestock attack), crops cultivated and the crops damaged by HBB, mitigation measures owned by local people, dependency on the forest, an approximate distance of the village from the nearest HBB habitat (habitat type we defined in our previous article, (Sharief et al., 2020)), distance from the road and the water resources were also recorded during the interview. The respondents' socioeconomic status was characterized using the Kuppuswamy socioeconomic scale, which accounts for education, occupation, and income together (Saleem, 2019). We used a one‐way analysis of variance (ANOVA) to understand whether the crop damage and livestock depredation varied significantly at different elevations and distances from the forest.

### 2.3 Spatial analysis of crop damage by the Himalayan brown bear (HBB)

Spatial analysis for crop damage by HBB in the study landscape was performed using SaTScan Ver 9.6, which is an extension of the scan statistics introduced in the 90s (Kulldorff, 1997; Kulldorff et al., 1998). The spatial scan statistics method was developed to analyze the localization, early detection, and for the substantial evaluation of clusters. The combination of the searching window and the resulting circle clusters build one of the most effective circle‐based scan statistics, which help to generate purely spatial, purely temporal or spatiotemporal hotspots by using the SaTScan software (Kulldorff, 2018). While in this study, we used purely spatial analysis with crop damage events of HBB and a discrete Poisson‐based model to run the analysis. This model helps to understand whether the reported spatial clusters are statistically significant or not and detect high or low rates cluster of damage events in the landscape. As per the null hypothesis, we assume the expected number of damages reported by interviewees in each village is in proportion to the total household in these villages. This model requires case and population counts for a given location, such as countries and zip code areas, and also needs the geographical coordinates for each of these locations (Kulldorff, 2018). We have considered the individual case counts for conflict as individual household reports and the total number of households within the village as the population count for the primary input file.

Furthermore, the spatial location of the villages acquired from the primary field survey was used as the location file in the SaTScan Ver 9.6. Then, we set the year as time precision because the analysis was purely based on spatial scale. The high or low rates for the scan areas with 9999 Monte Carlo replications and at least two cases were examined to restrict the high‐rate clusters with relative risk. The relative risk was defined as the estimated risk within the cluster divided by the estimated risk outside the cluster of a given area. It was calculated as the observed risk divided by the expected risk within the cluster and then divided by the observed divided by the expected risk outside the cluster. The mathematical equation to evaluate the relative risk (RR) is as follows:

$$\mathrm{RR}=\frac{\frac{c}{\frac{E\left[c\right]}{\left(c-c\right)}}}{\left(E\left[C\right]-E\left[c\right]\right)}=\frac{\frac{\frac{c}{E\left[c\right]}}{\left(c-c\right)}}{C-E\left[c\right]}$$

where, *c* = cases observed within the cluster and *C* = the total number of cases in the entire area. *E*[*C*] = total no. of expected cases in the entire area, *E*[*c*] = total no. of expected cases within the cluster since the analysis is conditioned on the total number of cases observed, *E*[*C*] = *C* (Kulldorff, 2018).

## 3 RESULTS

A total of 398 respondents (294 males and 104 females) were interviewed to understand the human–brown bear conflict on the livestock depredation, crop damage (agriculture/horticultural crops) and human casualties. The age of interviewees ranged from 15 to 88 years (average = 45.91). Among these respondents, about 71% of respondents admitted their dependency upon the forest for livestock grazing or the collection of nontimber forest products. Based on the information obtained, in the last 5 years, no human casualties were caused by a brown bear in Lahaul valley, but only two incidences where brown bear assaulted nomadic shepherds in the Lahaul valley during day hours. Whereas, two types of human–brown bear conflict in Lahaul valley were prominent viz., (1) crop damage (58.54%) (agricultural/horticultural) and (2) livestock‐depredation (34.42%).

### 3.1 Himalayan Brown bear conflict across socioeconomic classes

**FIGURE 3:**
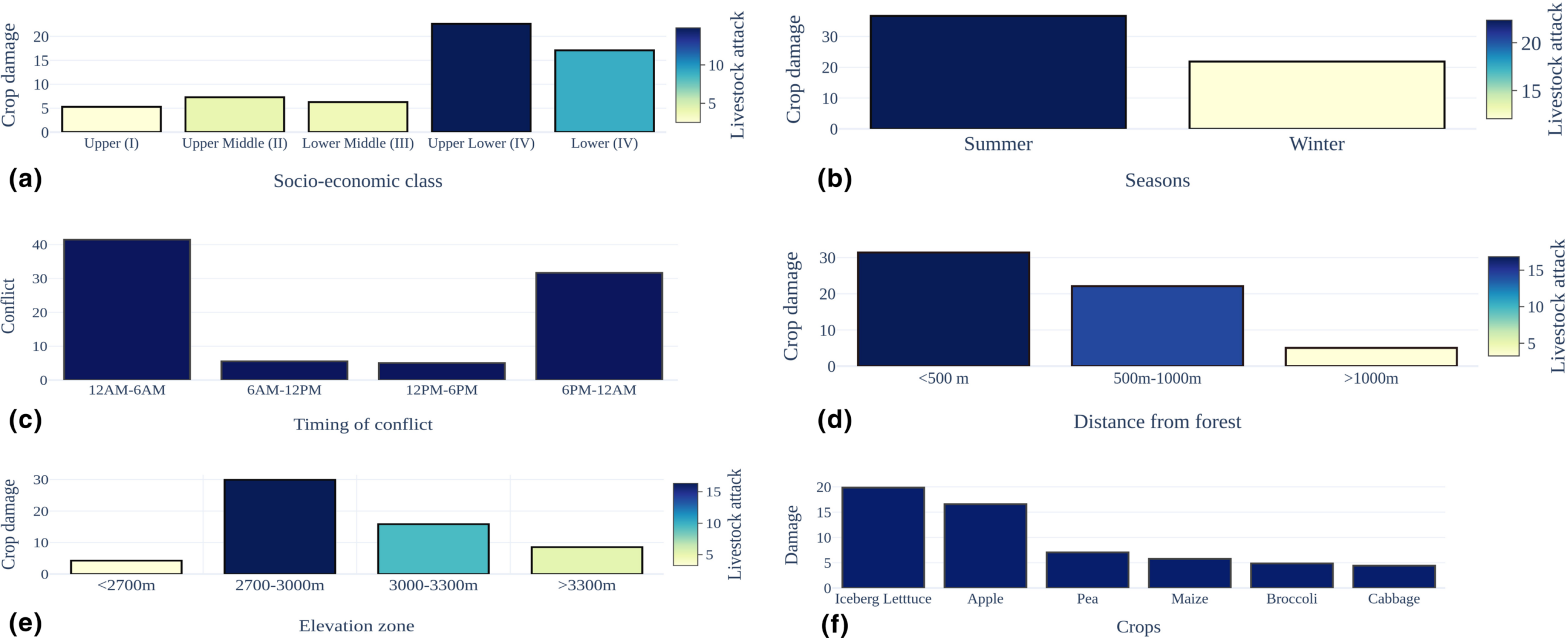
Depicts the human–brown bear conflict in the Lahaul valley. (a) showing socioeconomic class wise crop damage (Bar graph) and livestock attack (color ramp), (b) showing season wise crop damage (Bar graph) and livestock attack (color ramp), (c) showing timing of conflict in Lahaul valley as per respondent (*n* = 398), (d) showing crop damage (Bar graph) and livestock attack (color ramp) by HBB at different distance range from forest/brown bear habitat, (e) shows crop damage (Bar graph) and livestock attack (color ramp) at different altitudinal zone by HBB, (f) showing the main crop damaged by HBB in Lahaul valley

Based on the Kuppuswamy socioeconomic scale, of the total 398 respondents, the upper–lower class (IV) constitutes about 35.7%, followed by lower (V) (30.9%), and the upper (I) socioeconomic class constitute only 8.29%. Among these five socioeconomic classes, the crop and livestock damage because of HBB was in following order: upper–lower (IV) crop (22.61%) and livestock (14.82%) damage, followed by lower (V) (crop‐17.09%, livestock‐9.05%), upper–middle (II) (crop‐7.29%, livestock‐4.27%), lower–middle (III) (crop‐6.28%, livestock‐3.77%) and lowest one was of upper (I) (crop‐5.28%, livestock‐2.51%) (Figure 3). Furthermore, the ANOVA result suggests significant variation between the five socioeconomic groups for crop damage incidence (*p* = .045).

### 3.2 Crop damage by the Himalayan brown bear

In the Lahaul valley, the human settlements are closely located to the brown bear habitat, leading to a high level of crop damage. Among the crops, 19.85% of respondents admitted iceberg lettuce was the most depredated crop, followed by apple orchard (16.58%) (Figures 3 and 5). In which 36.68% of the crop depredation incidence was recorded during summer (Figure 3) followed by winter (21.86%) in the valley. The brown bear conflict mostly took place from midnight 100 h–600 h in the morning (41.46%), followed by evening from 1800 h to late‐night 2400 h (31.66%) (Figure 3). We found that the human–HBB conflict increases as the distance of cropland decrease from the nearest brown bear habitat. As the crop damage incidences were highest (31.41%) in locations with <500 m distance from forest/brown bear habitat, followed by 500–1000 m (22.11%) and >1000 m (5.03%), respectively (Figure 3). The conflict incidences also varied by altitudinal zone. However, the crop damage did not differ significantly (*p* > 1) among the elevation zones. Still, the highest number of incidences took place in elevation gradient of 2700–3000 m (29.90%), and the least was at localities <2700 m (4.27%; Figure 3). Moreover, the crop incidences also did not vary significantly with the distance from forested areas to the village (*p* > 1).

### 3.3 Livestock depredation by the Himalayan brown bear and protection measures used by locals

The data on livestock depredation suggested that among all livestock species depredated, the sheep, goats, and cattle were the most predated domestic animals by the brown bear. The livestock depredation was reported by 34.42% of the respondents, with the highest reports during summer (22.36%) than in winter (12.06%; Figure 3). The livestock damage also varied with distance from forest, with most of the cases taking place in areas <500 m (16.83%), followed by 500 m–1000 m (14.32%), and least in distantly located locations >1000 m distance (3.27%; Figure 3). The livestock depredation also varied significantly (*p* < .05) with altitudinal range, and the highest incidence took place in the elevation zones of 2700 m–3000 m (16.33%), followed by the 3000 m–3300 m (9.55%) (Figure 3).

Only 12.31% (49 of the total respondents 398) respondents use protective measures to protect their livestock and crops from getting depredated by HBB. These include electric or solar fences, scarecrows, barbed wire fencing in croplands, and some localities communities also use a metal container as a drum to scare the bear.

### 3.4 Spatial analysis of crop damage

**FIGURE 4:**
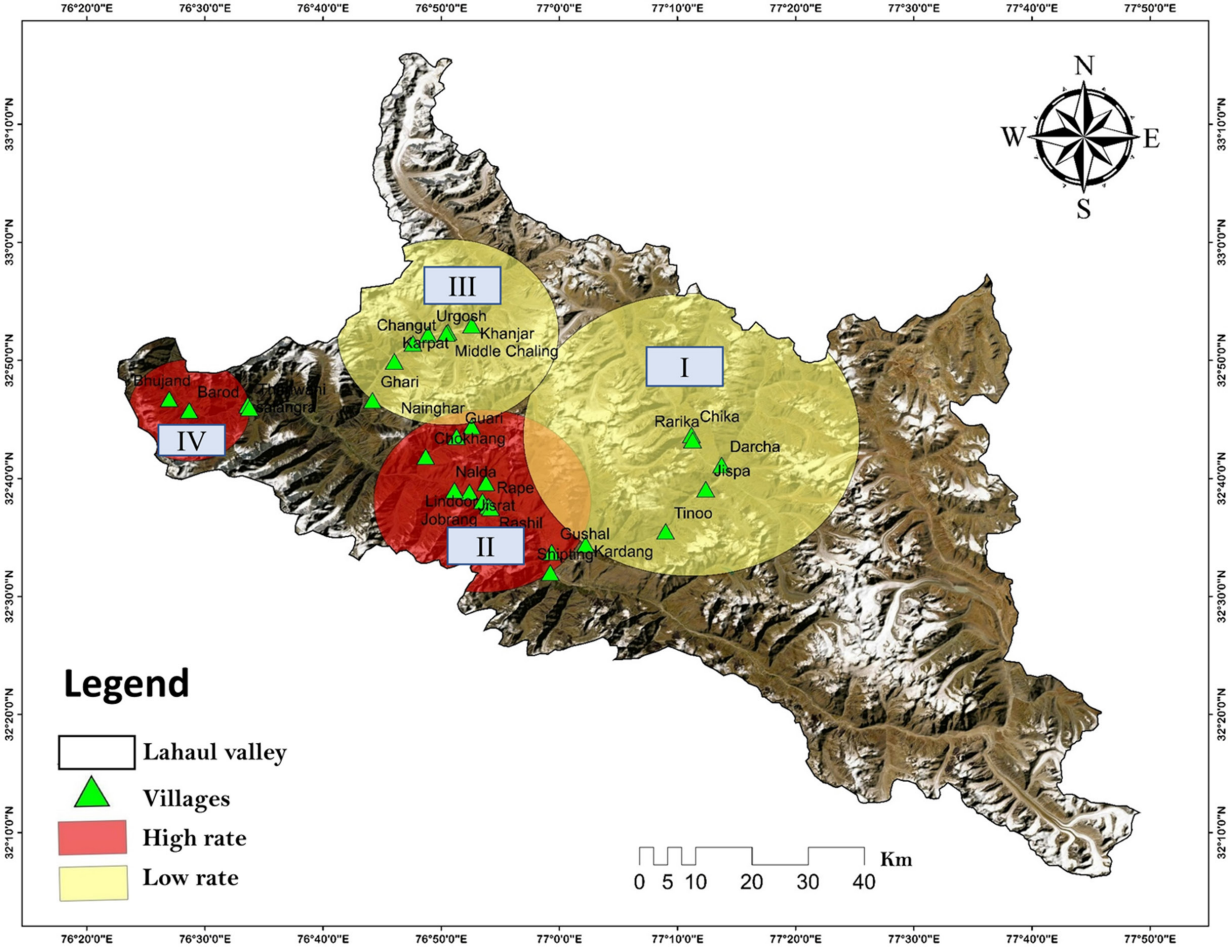
Depiction of crop damage by Himalayan brown bear in Lahaul valley. Red cluster shows the high rates of crop damage areas while yellow cluster depicts the low rates of crop damage by Himalayan brown bear

The spatial analysis results depict that cropland damaged by HBB form two clusters II and IV (Figure 4). The radius of the high‐rate clusters was 14.35 km (cluster II) and 7.97 km (cluster IV), with an observed/expected value of 1.6 and 1.58, respectively (Table 1). This suggests that the observed number of cases inside the clusters were higher than the expected number of cases. For clusters I and III, the observed/expected values were 0.07 and 0.65, respectively, which detects fewer observed cases than expected. Furthermore, the relative risk of conflict was higher in clusters II and IV with values of 2.22 and 1.69, respectively, and was lower in clusters I and III with values of 0.06 and 0.59.

**TABLE 1:** Trends of crop damage by Himalayan brown bear in the four clusters based on spatial model using SaTScan in Lahaul Valley

Cluster no	Locations	Coordinates	Radius (in km)	Observed/expected	Relative risk	*p*‐value
I	Chika, Rarika, Darcha, Jispa, Tinoo, Kardang	32.717 N, 77.167 E	22.18	0.07	0.06	0
II	Jobrang, Rape, Rashil, Jisrat, Lindoor, Nalda, Chokhang, Guari, Nainghar, Gushal, Shipting,	32.624 N, 76.872 E	14.35	1.6	2.22	.00
III	Chaling, Middle Chaling, Urgosh, Khanjar, Changut, Karpat, Ghari	32.862 N, 76.824 E	14.60	0.65	0.59	.80
IV	Barod, Bhujand, Salangra, Thanwani	32.752 N, 76.459 E	7.97	1.58	1.69	.92

In this table, locations refer to the name of villages in which questionnaire survey was made, in coordinates column show their GPS location and radius (km) depicts the radius of high‐ and low‐risk cluster of the conflict.

### 3.5 Respondent attitude and perception on Human–brown bear conflict

About 93% (*n* = 370) of the respondents affirm that they are impacted by HBB conflict (agricultural/horticultural crops or livestock depredation). About 83% (*n* = 334) of respondents did not appreciate the existing compensation policy of the forest department for the damage caused by the HBB conflict. When asked about their perception toward HBB, most the 75% (*n* = 302) dislike HBB in their locality because they lead to economic loss (crop damage and livestock depredation). About 56.28% of respondents reported that the easy access to crops by the HBB is the main reason for the increasing HBB conflicts, followed by poor natural food availability (11.55%) in the wilderness areas. Whereas, only 10.80% of respondents said natural calamities were behind increasing HBC cases, and 5.02% reported that habitat encroachment and fragmentation as the cause of increasing HBC in the valley.

## 4 DISCUSSION

**FIGURE 5:**
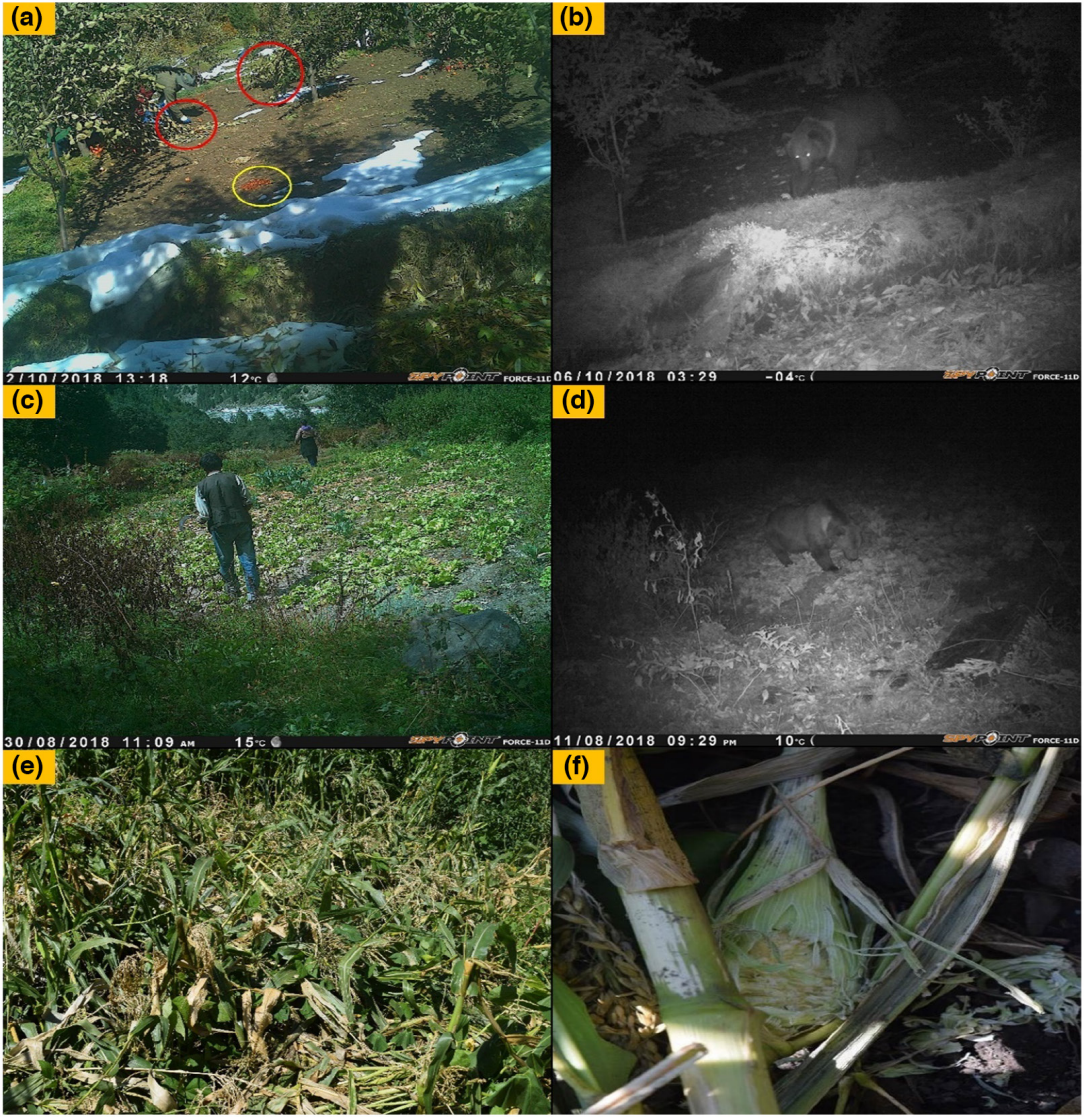
Camera traps/field images showing the incidences of crop depredation by HBB. (a) the camera trap image of apple orchard in which HBB damaged the apple tree, villager collecting the apple and HBB scat in yellow circle. (b) during the night at the same location HBB raiding the orchard. (c) Camera trap image of iceberg lettuce field and owner working in it. (d) at same site HBB captured during night depredating on iceberg lettuce. (e and f) maize fields photographs showing the maize filed damaged by HBB in Lahaul valley, Himachal Pradesh

The human–HBB conflict is identified as one of the major problems in ensuring the long‐term conservation of the brown bear in the Western Himalayas (Sathyakumar, 2001) and elsewhere (McLellan et al., 2017). The present study assesses the human–HBB conflicts in Lahaul Valley and the drivers of conflict in the landscape for conservation and management. Our results indicate (56.28% of respondents) that the agricultural and horticultural lands near forest areas attract bears due to the easy and high‐quality food available, which corroborates with the findings of other studies (Bargali et al., 2005; Can & Togan, 2004; Charoo et al., 2011; Rathore & Chauhan, 2014a, 2014b; Rigg et al., 2011; Steyaert et al., 2016). Furthermore, most of the crop damage by HBB was made during night hours in the absence of humans. This was also verified from our seven cameras of 12 placed near agricultural lands (Figure 5), and similar findings of the other studies conducted elsewhere (Lamb et al., 2020; Mace et al., 1996; Ordiz et al., 2011; Støen et al., 2015). The higher percentage of HBC (crop damage and livestock depredation) observed within the elevation zone of 2700–3000 m could be because the crop and grazing lands are distributed at this elevation and primarily distantly located from the human settlements. The expansion of agricultural lands and the adoption of high economic crops in place of traditional crops in the study landscape is one of the reasons for the increase in conflict cases in the study area.

Nevertheless, during our field survey, we noticed that the traditional cultivation of potato (*Solanum tuberosum*) is replaced by high economic crops (Iceberg lettuce, broccoli, red leaf lettuce, etc.), which attract HBB. Hence, these shifts in agricultural practices are playing a major role in increasing human–brown bear conflict in the landscape. The bears are attracted to the rancid smells of the rotten horticultural and agricultural crop residuals from long distances because of their extraordinary olfactory abilities (Green et al., 2012). The livestock depredation in high‐altitude areas of the study landscape could be because of unsupervised livestock grazing practices in alpine pastures by the nomadic herders. This is consistent with the results of our previous study (Sharief et al., 2020), which brought out that in Pattan valley with higher elevation villages such as Jobrang, Rashil, Goushal, Shipting, Mooling, Jasrath, and Nalda are more prone to brown bear conflict. The SaTScan results showed that cluster II, with a radius of 14.35 km, encompasses the villages mentioned by Sharief et al. (2020), resulting in higher crop damage and more cases than expected (Table 1 and Figure 4). Clusters II and IV cover the areas with a higher probability of HBB occupancy (Sharief et al., 2020) and have ideal habitats for HBB in the valley. Furthermore, HBB habitats are also in close vicinity of human settlements in these areas, and frequent migrations of the nomadic shepherd through these habitats during summers also increase the grazing pressure, degradation of habitats, and depletion of natural food resources (Baruch‐Mordo et al., 2014; Elfstrom et al., 2014; Sharma et al., 2021).

Consequently, HBB depredates readily available crops in agricultural lands that fall under cluster II and IV before hibernation to meet energy requirements during a hyperphagia period (Herrero, 2018). In the study landscape, human food is the main attractant for HBB, consistent with the other studies (Bojarska & Selva, 2012; Bombieri et al., 2019; Rathore, 2008). However, villages that fall under clusters I and III had few HBC instances due to fewer numbers of horticulture orchards, less scattered agricultural lands, and low detection probability of HBB in the areas.

In the valley, traditional protection measures were adopted to prevent damage, such as metal drumming and crackers, which were effective up to some extent. However, such practices may not have worked because of differences in localities. The respondent claimed only two incidents of lethal removal of HBB, suggesting retaliatory killing of the brown bear to reduce damage. Several studies have highlighted that the retaliatory killing of bears is a major conservation and management issue for bears in the Himalayas and elsewhere (Charoo et al., 2011; Rathore, 2008; Temesgen et al., 2022). The local communities have developed a negative perception of HBB due to HBC's‐associated high economic loss. Thus, it implicates a more significant threat to HBB conservation in the study landscape.

## 5 MANAGEMENT RECOMMENDATIONS

The HBC is a growing conservation and management issue in the study landscape. The local communities are developing negative perceptions about the bear's presence in the vicinity, which is alarming for the long‐term viability of HBB in the study landscape. The present study has identified a major human–HBB conflict hot spot, facilitating local stakeholders to identify high‐priority villages for conflict intervention. Furthermore, the identified hot spots will encourage mitigation efforts to focus on the vulnerable regions and provide suggestions to improve the current mitigation approaches cost‐effectively (Tripathy et al., 2021).

The local communities have adopted protective measures, including barbed wire fencing, metallic doors in cattle sheds, crackers to scare away bears from human settlements, and installed scarecrows at corners of the croplands. However, many of them are less effective in controlling the increasing instances of HBB in the landscape. Thus, to mitigate HBC in the study landscape, there is a need to adopt strategies such as installing electric fencing in HBC hot spots/ cluster II and IV village boundaries with the support of the Government of Himachal Pradesh by subsidizing the cost at the community level. These electric fencings effectively control the HBC elsewhere (Ambarlı & Bilgin, 2008; Huygens & Hayashi, 1999; Linnell et al., 1996). The garbage/ waste management mechanism may be considered for removing the crop residuals, which act as bear attractants in cluster II, where horticulture is more intense than in the other clusters. As anthropogenic food such as livestock feed, agricultural, and horticultural crops residuals attract brown bears (Bojarska & Selva, 2012; Herrero & Higgins, 2003). Creating community watch groups in the landscape can significantly minimize the crop damage where the community members should opt for turn‐wise guarding of agricultural crops and apple orchards (Charoo et al., 2011). Our results suggest that the local communities are least interested in conserving HBB in the landscape because of the economic losses. Therefore, appropriate compensation schemes for crop damage, crop insurance, and other conflict mitigation strategies must be implemented to change local attitudes.

Furthermore, change in cropping patterns such as cultivating crops such as potato, capsicum, and amaranths in place of maize and crops which attract bears may be adopted in croplands which are on the forest fringe. Ecotourism holds potential in changing the local's perception as reported in adjoining valley hence shall be promoted in Lahaul valley. The Lahaul valley has some of the most charismatic species, such as snow leopard, musk deer, and also brown bear, which will attract wildlife enthusiasts. Furthermore, ecotourism has significantly changed the lives of remote communities elsewhere and provided positive dividends toward improving the population of conservation‐dependent species such as snow leopard (Vannelli et al., 2019). Therefore, the involvement of locals and the nomadic shepherds in planning and implementing brown bear conservation actions in high conservation priority areas of Lahaul valley will be of significant importance. Furthermore, there is a need to notify few areas as protected areas identified in our previous study (Sharief et al., 2020), so that effective management actions related to habitat improvement, protection, and resource extraction can be minimized.

## AUTHOR CONTRIBUTIONS

**Vineet Kumar:** Conceptualization (lead); data curation (lead); formal analysis (lead); methodology (equal); visualization (equal); writing – original draft (lead); writing – review and editing (equal). **Amira Sharief:** Conceptualization (equal); data curation (equal); formal analysis (equal); methodology (equal); writing – original draft (equal); writing – review and editing (equal). **Ritam Dutta:** Conceptualization (supporting); data curation (equal); formal analysis (lead); investigation (supporting); methodology (equal); software (equal); writing – original draft (supporting); writing – review and editing (supporting). **Tanoy Mukherjee:** Formal analysis (equal); investigation (equal); methodology (equal); validation (equal); writing – original draft (supporting); writing – review and editing (supporting). **Bheem Dutt Joshi:** Conceptualization (supporting); data curation (supporting); formal analysis (supporting); investigation (supporting); methodology (equal); supervision (equal); writing – original draft (supporting); writing – review and editing (supporting). **Kailash Chandra:** Project administration (lead); resources (equal); supervision (equal); writing – original draft (supporting); writing – review and editing (supporting). **Mukesh Thakur:** Formal analysis (supporting); investigation (equal); project administration (equal); resources (supporting); supervision (equal); visualization (equal); writing – original draft (supporting); writing – review and editing (supporting). **Bhupendra Singh Adhikari:** Investigation (supporting); methodology (equal); project administration (supporting); supervision (equal); writing – original draft (supporting); writing – review and editing (supporting). **Lalit Kumar Sharma:** Data curation (supporting); funding acquisition (lead); investigation (equal); methodology (equal); project administration (equal); resources (lead); software (equal); supervision (lead); validation (equal); visualization (lead); writing – original draft (equal); writing – review and editing (lead).

## CONFLICT OF INTEREST

The authors declare no conflict of interests.

## Data Availability

The data will be provided on request.
